# Emergent Management of Intracardiac Thrombosis during Liver Transplantation

**DOI:** 10.1155/2016/6268370

**Published:** 2016-12-14

**Authors:** Caroline Protin, Dmitri Bezinover, Zakiyah Kadry, Thomas Verbeek

**Affiliations:** ^1^Department of Anesthesia and Perioperative Medicine, Penn State Health Milton S. Hershey Medical Center, Hershey, PA, USA; ^2^Department of Surgery, Penn State Health Milton S. Hershey Medical Center, Hershey, PA, USA

## Abstract

Intraoperative thromboembolism is a well-documented complication associated with orthotopic liver transplantation (OLT) but its identification and intraoperative treatment are still an emerging topic in anesthesia. Intracardiac thrombus during OLT is associated with a high mortality rate. There are only a few reports describing the successful management of thromboembolism during OLT. We describe a case where routine intraoperative transesophageal echocardiography during a live donor liver transplantation enabled early detection of an intracardiac thrombus with subsequent successful heparin treatment. Our case suggests that if an intracardiac thrombus is identified early (before hemodynamic instability occurs), the use of IV heparin may be a safe therapeutic option.

## 1. Introduction

End-stage liver disease (ESLD) is associated with an increased risk for both hypo- and hypercoagulopathy [1]. Hypocoagulability is relatively easy to detect and treat. Hypercoagulability, on the other hand, is frequently unexpected and difficult to diagnose before hemodynamic instability occurs. Once diagnosed, treatment is challenging and outcomes vary. Intracardiac thrombus (ICT) formation during orthotopic liver transplantation (OLT) is associated with a mortality rate as high as 68% [2]. A small number of published reports describe the successful use of recombinant tissue plasminogen activator (rTPA) as well as thrombectomy in the treatment of both intracardiac and pulmonary thrombi [3]. There are some reports of the use of intravenous heparin for the treatment of ICT during OLT in hemodynamically unstable patients [3]. All of these attempts failed. In this report, we describe a case where an intraoperative ICT was detected early in a hemodynamically stable patient and treated successfully with IV heparin.

## 2. Case Description

A 42-year-old female with a history of extrahepatic cholangiocarcinoma presented for a Living Donor Liver Transplant (LDLT). She was status post en bloc extrahepatic bile duct resection with right hepatectomy three years earlier with subsequent neoadjuvant chemotherapy and had developed high-grade recurrence. The patient's other pertinent past medical history included depression, asthma, and an episode of acute renal failure secondary to dehydration. The patient's only medications at the time of surgery were albuterol/ipratropium, cetirizine, and sertraline. Model for End-Stage Liver Disease (MELD) score was 25.

**Figure 1:**
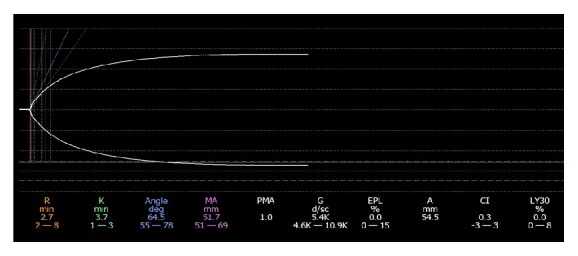
Thromboelastogram during preanhepatic phase after surgical incision.

Anesthesia was administered according to institutional protocol. In addition to standard American Society of Anesthesiologists monitoring, a transesophageal echocardiography (TEE) probe was placed after anesthesia induction. Coagulation was monitored using thromboelastography (TEG) and standard laboratory tests included International Normalized Ratio (INR), activated Partial Thromboplastin Time (aPTT), Prothrombin Time (PT), and fibrinogen concentration. The patient received 5000 units of subcutaneous heparin prior to anesthesia induction as routine deep venous thrombosis prophylaxis. Initial TEE exam performed after induction did not reveal any abnormalities. Standard midesophageal 4-chamber view was used for intraoperative monitoring. During the case, cardiac evaluation was performed every 20–30 minutes using midesophageal bicaval and midesophageal right ventricular inflow-outflow views, according to institutional protocol. All baseline standard laboratory tests including coagulation studies were obtained after induction and were within normal reference ranges. A TEG obtained during the preanhepatic phase was also within normal limits, with the exception of a *k* value that was mildly elevated (Figure 1).

**Figure 2:**
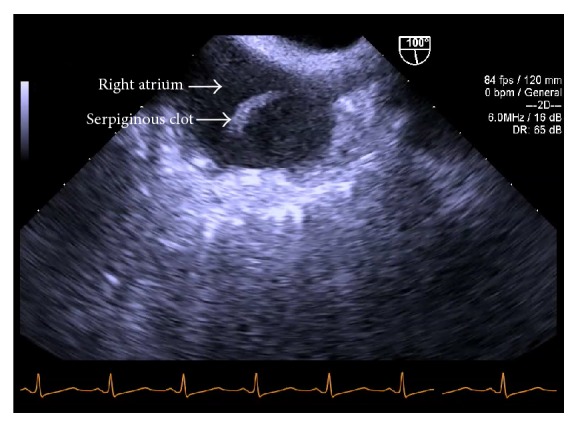
Preanhepatic phase. TEE midesophageal bicaval view: fibrous clot in right atrium.

**Figure 3:**
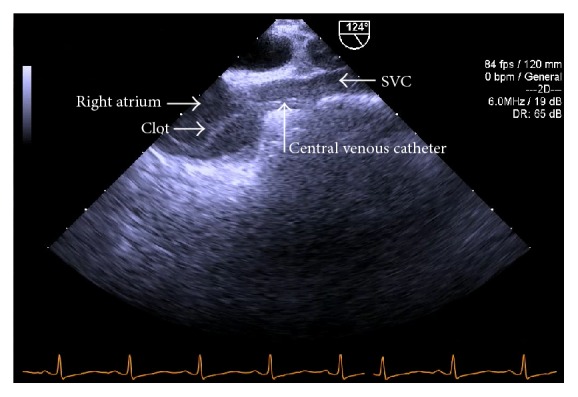
Preanhepatic phase. TEE midesophageal view: clot in the right atrium appears to be attached to 9 French catheter.

Six hours after induction, during dissection of the liver, the midesophageal bicaval TEE view demonstrated a mobile serpiginous fibrous clot, 2.5 cm × 1 cm, in the right atrium (Figure 2) which appeared to be attached to the 9 French catheter placed in the right internal jugular vein (Figure 3).

**Figure 4:**
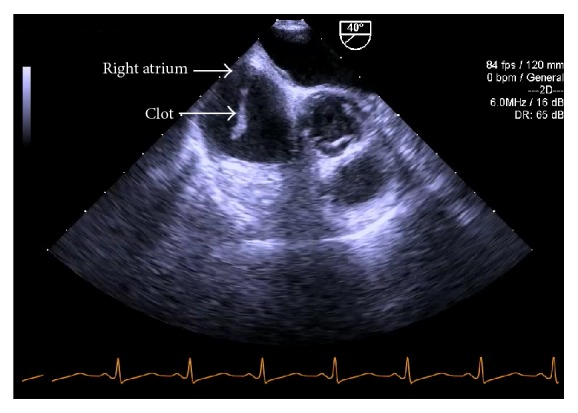
Preanhepatic phase. Modified TEE midesophageal right verticular inflow-outflow view: serpiginous clot in the right atrium.

We confirmed the presence of the clot using different TEE views (Figure 4). A discussion ensued between the surgical and anesthesia teams as to how to best proceed with the case. Options included the use of rTPA, cardiopulmonary bypass, thrombectomy, and IV heparin administration. Until this point, the patient had been hemodynamically stable. Point of care blood gas analysis did not reveal any abnormalities. Repeat TEE evaluation revealed a slight increase in clot size but the patient's hemodynamic status remained stable. Right and left ventricular function was not affected. The patient did not have any signs of increased pulmonary arterial pressure. Ultimately, it was decided to administer an initial bolus of 5000 units of IV heparin followed by a continuous infusion with a target aPTT of >60 seconds.

**Figure 5:**
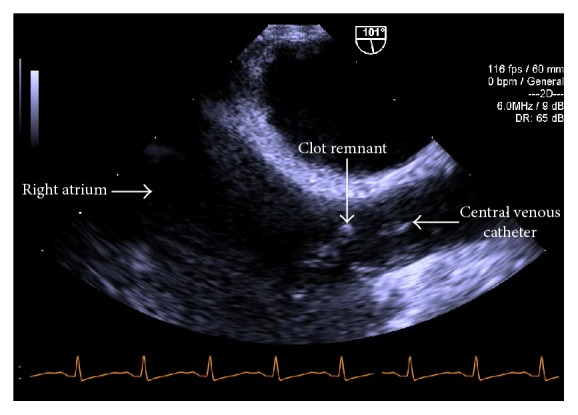
Neohepatic phase. TEE midesophageal bicaval view (depth decreased to 6 cm): significant reduction in clot size.

After graft reperfusion, TEE demonstrated a decrease in size of the clot and normal right and left ventricular function (Figure 5).

The patient remained stable throughout the case, requiring transfusion of two units of packed red blood cells (PRBC). The patient was transferred to the surgical intensive care unit postoperatively in stable condition and was successfully extubated on postoperative day (POD) 4. Graft function at that time was normal and there was no evidence of bleeding. The heparin infusion was continued postoperatively for 5 days after which anticoagulation therapy was transitioned to warfarin as soon as the patient was extubated and her surgical drains were removed.

A transthoracic echocardiogram (TTE) performed on POD 1 demonstrated a mobile, shaggy mass suggestive of a right atrial thrombus but was otherwise normal. There was no evidence of right heart strain. A repeat TTE on POD 9 was unable to demonstrate the presence of a clot in the right atrium and was also negative for any right heart strain.

## 3. Discussion

The incidence of intraoperative thrombosis in adult patients undergoing OLT ranges from 1.2% to 6.25% [4–6]. Eighty-five percent of thromboses occurred during the neohepatic phase of OLT and can pose a significant threat to both patient and graft survival [5, 7]. This hypercoagulability is related to complex changes in the coagulation profile seen in patients with ESLD. While there is a decrease in the synthesis and concentration of procoagulation factors by the liver, there is also a decrease in the synthesis and concentration of anticoagulation factors as well as a pronounced increase in production of liver-independent factors such as factor VIII, von Willebrand factor (vWF), and Plasminogen Activator Inhibitor 1 (PAI-1) [8–11]. Additionally, serum concentration of ADAMTS13, the protein responsible for splitting vWF, is decreased during OLT and its concentration does not immediately return to normal reference values after transplantation [11]. This results in a fragile coagulation/anticoagulation equilibrium that can easily be tipped towards bleeding or clotting.

Considering that our patient had a low MELD score but an oncologic etiology of her ESLD, she was predisposed to hypercoagulability. Vigilant monitoring is warranted in this patient population. The routine use of TEE during OLT can help detect clotting early; it also allows monitoring of cardiac function and guides management of hemodynamic instability should it occur. Shillcutt et al. demonstrated, in a retrospective study, that pulmonary microemboli were found in 44% of OLT patents. Twenty-seven percent of patients had thromboembolic events (24% intracardiac thrombus and 5% pulmonary thrombus) and the majority of these events occurred after graft reperfusion. ICT was associated with likelihood of early (30 days) and late (1 year) major postoperative adverse events and decreased survival (OR = 4.7, OR = 3.15, and OR = 3.46, resp.) [12].

The timely diagnosis of clotting events is crucial for intraoperative management. Several different methods to treat intracardiac thrombus formation and pulmonary thromboembolism previously have been described: IV heparin, rTPA, thrombectomy, and extracorporeal membrane oxygenation (ECMO). Heik et al. have demonstrated the effectiveness of high dose heparin administration for treatment of echocardiographically documented left ventricle thrombi but not in the OLT setting. In their report, the clots were significantly reduced in size or disappeared within 1 to 3 weeks without complications [13]. Boone et al. reported a case series of four patients where they used low dose rTPA (0.5 mg–4 mg) to treat intracardiac and pulmonary thrombosis during OLT [3]. In each of these cases, patients were hemodynamically unstable and in different stages of surgery. In two of the cases, a bolus of IV heparin was given with no change in hemodynamics. The administration of low dose rTPA in each of the four cases resulted in rapid hemodynamic improvement. In all of these cases, TEE was performed routinely allowing for rapid evaluation and treatment of thromboembolic events. All four patients had favorable postoperative outcomes.

Thrombectomy is another treatment option for intraoperative ICT and pulmonary embolus. However, while this method of intervention is more aggressive, it has not been demonstrated to improve patient outcome [2, 3, 14]. Other acute rescue therapeutic modalities such as ECMO or the Angiovac system (AngioDynamics, Latham, NY) have been used [15].

**Figure 6:**
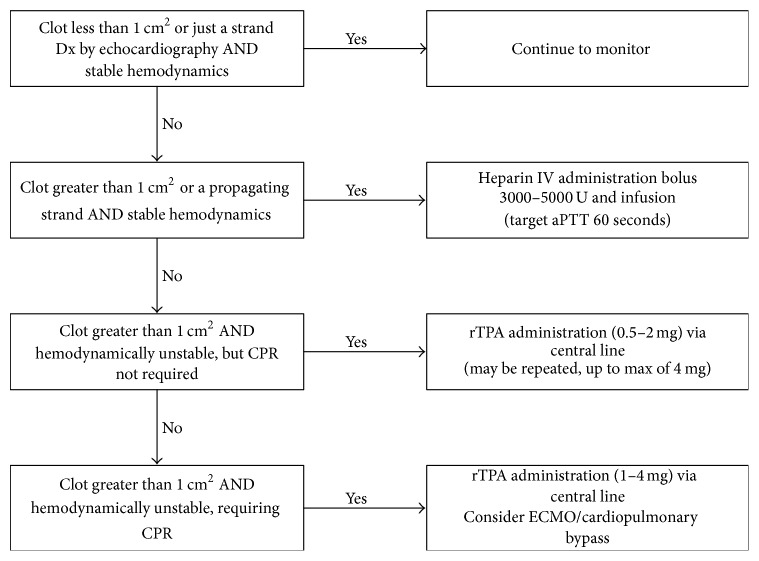
Management of an intraoperative intracardiac clotting event.

The use of TEE for routine monitoring during OLT is becoming commonplace in the USA. Considering that OLT is often associated with significant hemodynamic instability, intraoperative TEE is an important diagnostic tool for this patient population. It has been demonstrated that echocardiography used as a rescue modality can significantly improve outcome of patients with hemodynamic instability during noncardiac surgery [16]. Once a clot is detected, therapeutic options should be considered and immediately implemented. Based on our experience and current literature we established an algorithm in our institution for the management of ICT during OLT (Figure 6). The risk of bleeding complications may be reduced when low dose rTPA is used instead of the previously recommended dose of 100 mg for thrombolysis [3].

Our case suggests that the use of TEE may allow early detection of ICT prior to the development of hemodynamic instability. When identified early, intravenous heparin administration should be considered as a treatment option in this patient population as this approach may mitigate the need for more aggressive therapy.
